# Pregnancy management in laparoscopic promontofixation patients: a challenging situation with no clear recommendations

**DOI:** 10.11604/pamj.2020.36.248.21130

**Published:** 2020-08-05

**Authors:** Youssef Kharbach, Abdelhak Khallouk

**Affiliations:** 1 Urology Department, Mohammed VI Hospital, Faculty of Medicine, Abdelmalek Essaâdi University, Tangier, Morocco

**Keywords:** Pelvic organ prolapse, pregnancy, laparoscopic promontofixation

## Abstract

Uterine-sparing prolapse surgery offers fertility preservation; however, available data on the safety of pregnancy after surgery and the effects of pregnancy on surgical outcome are limited. Authors report herein reflections on the case of a 39-year-old woman with pelvic organ prolapse who underwent laparoscopic promontofixation. Pregnancy was diagnosed 2 weeks post-surgery. The main target of this research is to focus on pregnancy management before and after promontofixation due to the lack of data on the safety of pregnancy following surgery and the effect of pregnancy on surgical outcome. It seems preferable to us to operate patients in the first part of the cycle, if not after a dosage of beta-HCG and to provide effective contraception for at least 12 months.

## Introduction

Uterine-sparing prolapse surgery offers fertility preservation; however, available data on the safety of pregnancy after surgery and the effects of pregnancy on surgical outcome are limited [1]. Authors report herein the case of a 39-year-old woman with pelvic organ prolapse (POP) who underwent laparoscopic promontofixation. Evolutive pregnancy was diagnosed 2 weeks post-surgery.

## Patient and observation

A 39-year-old woman, para 5, was referred to us for exterior prolapse. She also complained of symptoms of stress urinary incontinence. Physical examination was performed. A stage 4 cystocele, stage 2 utero-vaginal prolapse and stage 3 rectocele were diagnosed. The therapeutic approach was a laparoscopic promontofixation using polypropylene meshes placed anteriorly in the vesicovaginal space and posteriorly to the levator ani. She was well-informed of the necessity to continue her oral contraception for 12 months after surgery. She came to us two weeks post-surgery with a positive beta-HCG. Actually, she had stopped her oral contraception and had an intercourse one week before promontofixation. She decided to continue with the pregnancy. The main target of this research is to focus on pregnancy management before and after promontofixation due to the lack of data on the safety of pregnancy following surgery and the effect of pregnancy on surgical outcome. We performed our bibliographic search on Pubmed and ScienceDirect with the keywords: “Pregnancy & Sacrohysteropexy,” “Pregnancy & Hysteropexy,” “Pregnancy & Uteropexy,” “Pregnancy & Colposacropexy,” “Pregnancy & Sacrohysteropexy,” as well as “Pregnancy & uterine sparing surgery”.

## Discussion

Due to demographic changes, there is a growing trend of POP in women of childbearing age [2], so, many patients desire definitive surgical management prior to family completion. Many techniques have been described for the surgical treatment of POP whether vaginally, abdominally or laparoscopically exist in the literature [3], but little is published on subsequent pregnancy [4]. Laparoscopic hysteropexy is the treatment of choice for patients wishing to retain fertility [1]. In practice, patients are advised to complete their families before any pelvic floor surgery, but in some cases this is impossible because of the impact of prolapse on sexual function [1]. The management of pregnancy after promontofixation is delicate, on the one hand we have a lack of knowledge about the impact of pregnancy on the surgical correction results of genital prolapse but also of the impact that surgery could have on pregnancy, on the other hand, no academic society has issued a clear recommendation regarding the possibility of pregnancy after surgery or the need for contraception in order to avoid it. If it is accepted that a patient who is pregnant should not be operated on, it seems paradoxical to us that none of the academic societies recommend a pregnancy test before surgery or other precaution to avoid operating on a pregnant patient [5-7].

Nevertheless, we found in our literature review two cases of successful laparoscopic correction of uterine prolapse performed at the 10^th^ and 12^th^ weeks of gestation suggesting that it could be a safe technique during pregnancy [8]. If some authors say that surgery should only be done after childbearing is complete [9], others propose to patients still of childbearing age, undergoing uterine preservation surgery, an enlightened explanation of the possible consequences of pregnancy and offer them reliable and definitive contraception at the time of surgery (coil or salpingectomy) [1]. There are also no clear recommendations on obstetric management of women with pregnancy after promontofixation. However, there is a tendency not to encourage vaginal delivery and to prefer scheduled caesarean section [1-4]. We also don´t know which surgical technique is the most suitable and safe for patients wishing to conceive. Unfortunately, there is not enough data to support the safety and efficacy of different surgical approaches [3]. It seems reasonable to conduct a multi-centric study, and pool data in order to have robust evidence-based guidance. Pending these recommendations, it seems preferable to us to operate patients in the first part of the cycle, if not after a dosage of beta-HCG and to provide effective contraception for at least 12 months.

## Conclusion

Surgical treatment of POP is possible in women considering pregnancy. But, academic societies need to come out with clear recommendations to prevent surgical interventions on pregnant patients, define an optimal time after surgery to consider a pregnancy and possibly the best intervention to be proposed.
